# COVID-19 Mental Health Stressors of Health Care Providers in the Pandemic Acceptance and Commitment to Empowerment Response (PACER) Intervention: Qualitative Study

**DOI:** 10.2196/35280

**Published:** 2022-03-22

**Authors:** Christa Sato, Anita Adumattah, Maria Krisel Abulencia, Peter Dennis Garcellano, Alan Tai-Wai Li, Kenneth Fung, Maurice Kwong-Lai Poon, Mandana Vahabi, Josephine Pui-Hing Wong

**Affiliations:** 1 Factor-Inwentash Faculty of Social Work University of Toronto Toronto, ON Canada; 2 Daphne Cockwell School of Nursing Ryerson University Toronto, ON Canada; 3 Regent Park Community Health Centre Toronto, ON Canada; 4 Committee for Accessible AIDS Treatment Toronto, ON Canada; 5 Department of Psychiatry Faculty of Medicine University of Toronto Toronto, ON Canada; 6 School of Social Work York University Toronto, ON Canada

**Keywords:** COVID-19, COVID-19 in Canada, health care providers, pandemic stressors, health impact, caregiving roles, situational identities, emotional labor, hero discourse, social ecological framework

## Abstract

**Background:**

Since the pandemic, more Canadians have reported poorer mental health. A vital group experiencing a high level of stressors consists of health care providers (HCPs) caring for COVID-19 patients, carrying out public health responses, or working with vulnerable populations. The mental health of HCPs is negatively affected by the pandemic, not only at work but also at home and in the community. Intersecting stressors at multiple levels contribute to HCPs’ experiences of fatigue, insomnia, anxiety, depression, and posttraumatic stress symptoms.

**Objective:**

The aim of this qualitative study was to explore the pandemic stressors experienced by HCPs at work, at home, and in the community before participating in the Pandemic Acceptance and Commitment to Empowerment Response (PACER) online intervention.

**Methods:**

Informed by a social ecological approach, we used a qualitative reflective approach to engage 74 HCPs in diverse roles. Data were collected during the first 2 waves of the COVID-19 pandemic (June 2020 to February 2021) in Canada.

**Results:**

Informed by a social ecological framework, 5 overarching themes were identified in our thematic analysis: (1) personal level stressors that highlight HCPs’ identities and responsibilities beyond the workplace; (2) interpersonal level stressors from disrupted social relationships; (3) organizational stressors that contributed to unsettled workplaces and moral distress; (4) community and societal stressors attributed to vicarious trauma and emotional labor; and (5) the multilevel and cumulative impacts of COVID-19 stressors on HCPs’ health.

**Conclusions:**

COVID-19 is not merely a communicable disease but also a social and political phenomenon that intensifies the effects of social inequities. Current understanding of pandemic stressors affecting HCPs is largely partial in nature. Although workplace stressors of HCPs are real and intense, they need to be explored and understood in the context of stressors that exist in other domains of HCPs’ lives such as family and community to ensure these experiences are not being silenced by the “hero” discourses or overshadowed by professional demands.

## Introduction

In Canada, the COVID-19 pandemic has claimed over 31,000 lives [1]. It has profoundly affected the lives of Canadians, particularly their mental health and well-being. Although everyone is affected by the pandemic, the degree of COVID-19 impact varies across groups and communities. Since the pandemic, more Canadians have reported poorer mental health, particularly women, individuals with chronic illnesses or disabilities, racialized persons, immigrants, and Indigenous peoples [2,3]. A vital group experiencing a high level of stressors consists of health care providers (HCPs) directly involved in caring for COVID-19 patients, carrying out public health responses, or working with vulnerable populations. Emerging evidence in Canada and elsewhere indicates that the mental health of HCPs is negatively affected by the pandemic. HCPs have reported sleep disturbances, fatigue, and episodes of insomnia [4,5] as well as increased prevalence of anxiety [6,7], depressive symptoms [8,9], suicide ideation [8], and posttraumatic stress symptoms triggered by repeated intense stressful experiences and reduced social life [5,10].

During a pandemic, the popular image of HCPs is often limited to individuals in personal protective equipment (PPE) and saving lives. However, behind the masks and full PPE gear, HCPs are people with many social identities, roles, relationships, responsibilities, emotions, and needs. There is relatively limited qualitative research on the experiences of stress and challenges that negatively affect the mental health of Canadian HCPs, particularly those that extend beyond their professional identities and roles. A study with 20 Canadian nurses identified uncertainty, inadequate access to PPE, repeated witnessing of patient deterioration and death, and experience with social isolation as key sources of stress [11]. Two other studies found that HCPs experience increased stressors related to rapidly changing policy, unclear or lack of leadership communication, anxiety about safety for self and family, and tension between meeting the needs of their patients and pandemic protocols [9,12]. Studies in other countries also revealed similar multilevel stressors that affect HCPs’ mental health. Additional stressors include caring for and losing one’s own infected colleagues to COVID-19, inconsistent guidelines on COVID-19 testing and treatment, ineffective staff redeployment, and staff shortages [13-15]. All these intersecting challenges also contribute to moral distress, which can be defined as the emotional, psychological, and physical suffering that HCPs experience when they carry out their responsibilities in ways that are inconsistent or contradictory to the ethical values, principles, or commitments that they hold deeply [16].

Current understandings of pandemic stressors affecting HCPs are largely partial in nature. Since the beginning of the COVID-19 pandemic, HCPs (especially nurses) have been hailed as “unsung heroes” by politicians, the mass media, and the general public. In their poststructural analysis of over 70 documents in mass media and social media on “nurses as heroes,” Mohammed and colleagues [17] argued that the hero discourse positions nursing as “model citizens” and “a necessary sacrifice,” whereby heroism is the reward. When taken together, the hero discourse imposes a new form of subjectivity and normalizes nurses’ exposure to workplace risks. We further argue that the hero discourse has created a spotlight that solely focuses on the professional identity and roles of HCPs, negating the multiple identities that constitute their “holistic self” and their everyday lived experiences that are inseparable and indistinguishable in their intertwined home-work-community life. Thus, while workplace stressors of HCPs are real and intense, they need to be explored and understood in the context of stressors that exist in other domains of HCPs’ lives. In the context of a pandemic, stressors can be understood as an ever-present condition that threatens the health or well-being of individuals [18].

To date, an overwhelming majority of empirical studies on pandemic-related stressors among HCPs are quantitative in nature and report specific measurable aspects of stressors that are often decontextualized. Qualitative research on this topic is needed to provide insights on the multilevel contexts of how HCPs experience and are impacted by COVID-19 stressors. In this paper, we report on the qualitative data collected from HCPs who self-identified as experiencing a high level of stress and wished to enroll in an online stress-reduction intervention designed for HCPs and affected communities. The results presented contribute to addressing the gaps in our current understanding of pandemic stressors experienced by HCPs in the Canadian context.

## Methods

### Study Setting: The PACER Intervention

The Pandemic Acceptance and Commitment to Empowerment Response (PACER) intervention [19] is an evidence-informed 6-week online training based on the Acceptance and Commitment to Empowerment (ACE) model. ACE is an innovative integration of the mindfulness-based Acceptance and Commitment Therapy (ACT) [20] and the social justice-based Group Empowerment Psychoeducation (GEP) [21]. It has been applied effectively in previous in-person interventions to reduce stigma and promote resilience. Recognizing the growing mental health needs of frontline HCPs and constrained by pandemic lockdowns, we developed the PACER intervention, which consists of 6 weekly online self-guided learning modules, each lasting 1 hour, accompanied by weekly 90-minute facilitated online group video-conferencing sessions. Prior to engaging in the first online learning module and first group sessions, participants were invited to complete a sociodemographic survey and questionnaires that consisted of both quantitative and qualitative questions (see the Procedure section).

### Ethical Review

This study was reviewed and approved by the Research Ethics Boards at Ryerson University (2020-123), University Health Network (20-5210), and York University (2020-096).

### Participants

We used convenience and snowball sampling, supported by targeted outreach and recruitment via circulations of e-flyers through our collaborators, nursing professional associations, hospital bulletins, community networks, and social media. The participation criteria were self-identifying as 18 years of age or older, being a service provider in the health care sector, experiencing high levels of stress related to the COVID-19 response, and interested in joining the PACER training. HCP participants in this study included registered nurses (RNs), registered practical nurses (RPNs), personal support workers, physicians, medical residents, social workers, counsellors, therapists, and other clinical or community-based personnel.

A total of 74 HCPs enrolled and took part in PACER between June 2020 and February 2021: 63 (85%) self-identified as female, 10 (14%) self-identified as male, and 1 (1%) self-identified as transgender. Most participants were under 40 years of age: 20-29 years: 10/74, 14%; 30-39 years: 38/74, 51%; 40-49 years: 14/74, 19%; 50-59 years: 9/74, 12%; and 60-69 years: 3/74, 4%. When asked about the ethnocultural communities they identified with, about one-half of the participants (38/74, 51%) indicated that they identified with the Canadian community, while the other one-half (36/74, 49%) identified with multiple communities including African, American, Chinese, Filipino, Haitian, Irish, Hong Kong, Taiwan, Jewish, and Latinx. Participants came from a wide range of health and social care professional backgrounds and roles such as primary care, psychiatry, nursing, social work, public health, community health practitioners, therapists/counsellors, administrative staff, environmental service staff, and volunteers.

### Procedure

Before data collection, online informed consent was obtained from all participants. Once enrolled, participants were invited to complete a pre-intervention questionnaire that consisted of sociodemographic questions and measures on general mental health distress, psychological flexibility, and resilience. These measures were repeated immediately post- and 3 months postintervention. In addition, prior to Session One of the intervention, we used qualitative open-ended questions to explore participants’ self-identities and experiences with pandemic stressors. Specifically, we invited participants to describe “who you are” and “the impact of COVID-19 on your work and your life—what has been challenging for you.”

### Analysis

The focus of this paper was on the pandemic-related stressors reported by participants in the qualitative questions before they engaged in the 6-week online intervention and online group sessions. Responses from the online questionnaires were downloaded, and pseudonyms are used in this paper to protect participants’ identities. Our thematic analysis was informed by the social ecological framework [22], which allowed us to examine the sources of stressors experienced by HCPs at multiple interconnected levels [23]: personal (identity, social position), interpersonal (family, relationships, social networks), organizational (operational characteristics, formal and informal rules), community contexts (relationships among organizations and institutions), and societal (local, provincial, and national guidelines and policies) [24]. This approach enabled us to make sense of how different synergistic factors and contexts intersect to shape the experiences with pandemic stressors among HCPs.

The first 4 authors, under the guidance of the last author, conducted thematic analysis [25] using both inductive and deductive approaches. First, we engaged in repeated readings of the participant responses to gain a broad understanding of the data. Second, we identified and developed thematic codes inductively based on the ideas and perspectives articulated by the participants (ie, data-based coding). Third, guided by our research purpose and the social ecological framework, we applied deductive analysis that enabled us to connect the data to the sociocultural contexts and structural conditions that shaped the experiences of the participants [26,27]. Fourth, the team engaged in multiple rounds of interpretive discussions to arrive at the agreed themes presented in this paper. In the last round of thematic interpretation, all authors took part to discuss the nuances and contexts of the narratives under each theme to arrive at a holistic interpretation.

## Results

Informed by a social ecological framework, 5 overarching themes were identified in our thematic analysis: (1) personal-level stressors that highlight HCPs’ identities and responsibilities beyond the workplace, (2) interpersonal-level stressors from disrupted social relationships, (3) organizational stressors that contributed to unsettled workplaces and moral distress, (4) community and societal stressors attributed to vicarious trauma and emotional labor, and (5) the multilevel and cumulative impacts of COVID-19 stressors on HCPs’ health.

### Personal Stressors: Identities and Responsibilities Beyond the Workplace

Stressors at the personal level emphasize the identities and responsibilities of HCPs above and beyond the workplace that participants encountered in their day-to-day lives. Many participants expressed fear and worries about bringing the SARS-CoV-2 virus home to their elderly family members or loved ones with existing health problems.

My husband had been undergoing intensive chemotherapy and radiation treatment at [a cancer care hospital], and was halfway through a year of treatment... I was very scared that I might contract the virus at work and bring it back to our house... and impact his access to his next treatment.

Sharon, 30-39 years, female, children’s rehab coordinator, Canadian

It is also important to note that HCPs are not only service providers; some relied on accessing services to meet their own health needs or the needs of their family members. Hence, service disruptions are not only felt professionally but also personally at home or in their communities.

As the parent of a child with dual diagnosis I already feel isolated from society. My son is not capable of wearing a mask and as a result we have not been to any activities together since the first lockdown... My son does not understand the restrictions and has been suffering from lack of social interaction and engagement in his community.

Kathy, 30-39 years, female, behavior therapist, Canadian

To meet the demands of their multiple roles and responsibilities, some participants had to make use of available resources to mitigate some of these struggles. Sun, an RN, had to rely on her elderly in-laws to care for her toddler while she was at work. Meanwhile, she also expressed her concerns:

[We] fear the potential transmission of COVID-19 to my in-laws as they are of higher risk from pre-existing medical conditions and advanced age.”

Sun, female, 30-39 years, RN and case manager, Chinese-Canadian, Hong Kong

Sun’s fear was echoed by other HCPs living in multigenerational households comprised of vulnerable elderly adults and young children.

### Interpersonal Stressors: Disrupted Social Relationships

Similar to all Canadians, many participants identified disruptions to routines and structures in their personal life as stressors. Social distancing and quarantine measures had heightened participants’ sense of isolation and loneliness where social relationships are vital resources in fostering HCPs’ resiliency. For participants with many demands and responsibilities, travelling is not merely a leisure activity but a coping strategy to reduce stress. Andrew, who had to manage demands from school, work, and family, indicated that his once-a-year vacation was “a breather” from his stressful life. He further explained:

The vacation time is my only chance to see my family, who lives in the Philippines. As the pandemic unfolds, all my plans are put on hold.”

Andrew, male, 30-39 years, RPN in retirement facility, Filipino-Chinese

For participants with transnational family and social ties, pandemic travel restrictions resulted in prolonged separation and disconnection from their families. Those with elderly or sick family members felt additional challenges.

The biggest issue was, my mother is in advanced Alzheimer's back in India and her health had started to decline. Prior to [the] COVID-19 outbreak, I had booked a flight for India, which of course got cancelled.

Priya, female, 40-49 years, RPN in geriatric care facility and BScN student, South Asian

Furthermore, while the pandemic had forced many participants to put their plans on hold, it had not stopped other critical events from taking place around the world. Participants with families and loved ones in Iran, India, and the United States expressed a sense of powerlessness and worries due to the pandemic situation worldwide.

I have felt very helpless while watching the effects of COVID tear through the U.S., on top of all the other crises that the country is currently facing. I spend a lot of time worrying about my parents and I have had difficulty focusing on my schoolwork.

Adam, male, 20-29 years, graduate student in clinical psychology, American

Participants’ sharing illustrated that, in addition to their demands as frontline HCPs, they also shouldered many demands similar to the general population, even though these stressors were overshadowed by discourses of their professional demands.

### Organizational Stressors: Unsettled Workplace and Moral Distress

Not surprisingly, HCP participants encountered many workplace stressors, ranging from increased health risks related to direct exposure to COVID-19, changes in work routines and workloads, and the need to wear PPE all day. At the beginning of the pandemic, uncertainty about COVID-19 created a lot of fear among HCPs, particularly those with less formal biomedical or health care training. Matthew, an environment service staff member in a hospital, expressed tremendous fear toward COVID-19.

When I was assigned to clean rooms where there were patients with COVID-19... I was too scared to communicate with the patients. I limit our conversation by just answering yes or no. I feel very guilty because of my fear... I am working again tomorrow, I hope I will not encounter a patient with COVID.”

Matthew, male, 30-39 years, environmental service staff, Filipino

For some HCPs, their fear was not limited to personal safety but extended to the uncertainty and demands of keeping their staff and patients or vulnerable service users safe.

At times I feel overwhelmed that my support is not sufficient in supporting a large team. Stressed by the ongoing changes, angry at the impact it’s having on the homeless community and the lack of acknowledgement frontline staff in shelters and homelessness services receive.

Tracy, female, 40-49 years, community practitioner, Canadian

The rapid changes in the workplace routines and structures also led to considerable distress that altered or strained participants’ interpersonal relationships with their patients, colleagues, and other staff. For Kathy, a behavior therapist working with children diagnosed with autism spectrum disorder, it was difficult to carry out her work with PPE:

I miss being able to high five my clients and look into their eyes unobstructed by PPE in order to read the small changes in their expressions.

Kathy, female, 30-39 years, behavior therapist, Canadian

Other HCPs were concerned about access to safe PPE due to previous experience of receiving defective PPE.

Many participants identified deployment as a major source of stress. One RN experienced anger and resentment “when colleagues were redeployed to other areas of the hospital,” but her team was “expected to maintain the same caseload with less human resources” [Sun, female, 30-39 years, RN, Chinese-Canadian, Hong Kong].

Marianna, who was deployed to work in nursing homes, recalled the stress and exhaustion:

It was a bit stressful to learn 3 different positions within 3 months... I often worked overtime and weekends, so it left long stretches of very little time for myself and my family.

Marianna, female, 40-49 years, RN, Portuguese Canadian

In addition to deployment and increased workload, some HCPs experienced moral distress when their efforts to provide safe and quality care to their vulnerable patients were compromised by the rapidly changing policies at work, as Giselle explained:

Most of our patients have a history with mental health challenges and trauma. While my workplace is based on a harm reduction philosophy, it felt like we were also re-traumatizing our patients due to the new COVID-related policies.

Giselle, female, 20-29 years, RN, Filipino

Similarly, Ariana expressed her moral struggles and dilemma in attending to the special needs of families in her clinic.

Families… having their kids with behavior and mood challenges at home with them, much reduced access to in-person and out-of-home respite and other services… I am the point person for families… Ethical dilemma of having to medicate children to keep everyone sane and safe when we know it’s the circumstances brought about by COVID-19 restrictions that is making things worse.

Ariana, female, 50-59 years, clinical nurse, Canadian, Welsh

Other participants expressed concerns about workplace opacity and suggested that their employers had used pandemic emergency protocols to implement “different priorities” without consulting staff and patients. When participants’ concerns about safety and accountability toward patient care were dismissed, they were left feeling undervalued and disempowered.

When feedback was provided to management in regards to concerns about safe practice and lack of proper transfer of accountability between teams… I was met with ridicule and publicly humiliated, which caused me to feel insignificant, with feelings of powerlessness.

Sun, female, 30-39 years, RN and case manager, Chinese-Canadian, Hong Kong

As the HCP participants encountered stressors of an unsettled workplace, many also struggled with the moral distress of weighing the quality of care they could realistically provide to patients against the safety of self and others. Other stressors included guilt for minimizing interactions with clients for fear of contracting the virus, the impact of services on staff and clients working with vulnerable populations, or feeling dismissed when concerns about workplace policies and practices are brought to the attention of administrators.

### Community and Societal Stressors: Vicarious Trauma and Emotional Labor

Another stressor faced by HCPs during the pandemic is the intensified emotional labor placed on them at work. There seemed to be a collective resonance of vicarious trauma expressed by participants who witnessed the suffering of their patients or service users.

Witnessing the impact of Covid-19 on seniors and feeling helpless has a negative impact on my own mental health. One lonely senior passed away and one senior's cat was missing, that especially hit hard on mental health.

Margaret, female, 50-59 years, community health promoter, Chinese-Canadian, Mainland China

Amy, who witnessed and experienced the collective suffering, expressed her feelings of sadness:

Clients are dying alone or with the touch of a gloved hand; loved ones are not able to say their goodbyes; and questions are not always answered… makes me feel drained emotionally and physically… and it has made me feel guilty.

Amy, female, 30-39 years, RN and NP student, African-Canadian

As caregivers, HCPs often carried the emotional “burdens” of suppressing their own feelings while supporting and reassuring their loved ones, colleagues, patients, and service users who were in distress. Some of them also experience vicarious trauma as they witnessed the inequities and social oppressions experienced by individuals, families, and communities they worked with.

It has been devastating to see the inequity and injustice around, who has been most impacted by the pandemic, and this is evident in the families I support. Community resources are stretched and limited so there are less options for support. This has resulted in higher demands on our service. All of this combined has left me feeling more depleted and less hopeful.

Julia, 40-49 years, social worker of children living with disabilities, Canadian

Peggy, a Chinese-Canadian, described the pain in witnessing the most marginalized in the Asian Canadian community be further marginalized during the pandemic, due to “loss of income, mental health deterioration from social isolation, school closures, and experiences of anti-Asian racism” [female, 30-39 years, youth mental health clinician].

The demands of pandemic challenges were also felt by participants in management positions. Some of them felt inadequate when they were faced with concerns about the psychological and physical safety of both their staff and patients. This emotional labor could be difficult to bear when their efforts were met with abusive behaviors from the service users, as Louisa indicated:

Over the course of the past several months, I have been subject to ongoing verbal abuse by patients and families, who disagree with the COVID-19 related precautions that have been implemented.

Louisa, female, 30-39 years, hospital clinical manager, Canadian

Other HCPs reported experiencing overwhelming feelings of anger, frustration, worry, sadness, confusion, loneliness, helplessness, and burnout. Sun, who worked as a case manager, recalled:

...there was a period between April and June where I felt overwhelmed and started experiencing bitterness and resentment toward being a health care provider.

Sun, female, 30-39 years, RN, Chinese-Canadian, Hong Kong

Despite the overwhelming emotional demands and sense of uncertainty, some participants were also inspired by a sense of collectivism. Many commented on the strength they received from sharing a strong sense of team spirit that brought value and meaning to their work.

The collaboration and teamwork! The all-hands-on-deck approach to my organization's response to the pandemic. It was not as smooth, but the teamwork was visible in ensuring that staff are aware of policy changes or updates.

Julian, male, 30-39 years, RN, Filipino-Canadian

Other HCPs derived their sense of strength and optimism by focusing on “being part of a team working toward a greater goal, that we are all in this together, and a shared understanding of what it is to be a front-line worker during a pandemic” [Kamal, female, 30-39 years, intensive care unit nurse and critical care research coordinator, Canadian, Indian]. Hence, despite all the challenges encountered by HCPs, there were factors and contexts that promoted a sense of togetherness and connection with others, which can help foster resilience.

### The Multilevel and Cumulative Impact of COVID-19 Stressors on Health

As highlighted in the previous themes, participants experienced multiple ongoing and compounding pandemic stressors in the domains of work, home, and society that undoubtedly impacted their physical, psychological, and emotional health and well-being. These impacts can also be considered stressors in and of themselves because dealing with the impact of these challenges simultaneously contributes to secondary stressors.

COVID-19 and the quarantine process has led to a collective trauma, even those whose physical health is functioning, but they are impacted emotionally… I have had moments of wanting to end my life back in April.

Jess, gender-fluid, 30-39 years, health discipline student, Canadian, Hong Kong

Others described a range of physical problems from feeling drained, tired, burned out, concerns regarding sleep, poor concentration, and feeling tension and pain in their bodies. In some cases, individuals had pre-existing health conditions that worsened during the pandemic.

I am currently undergoing physiotherapy because I suffered a hip injury two years ago. Closure of the facility where I receive physiotherapy services [has] been a significant factor why I experience pain while I work. I only rely on my daily stretches and pain medications to cover-up the anticipated pain that I will experience at work.

Matthew, male, 30-39 years, environmental service staff, Filipino

For some HCPs, deployment and workplace chaos had taken a toll on their mental health, yet they felt guilty and blamed themselves.

When I was redeployed to an entirely new department my anxiety intensified, to the point that I needed to seek accommodation to work from home. During this time I had negative thoughts about myself, including thoughts that I was weak, that I was letting my colleagues down, or that I should be able to handle this better.

Julia, 40-49 years, female, social worker in field of disabilities, Canadian

Other HCPs prioritized the needs of others, sometimes to their own detriment because they neglected their own emotional and psychological needs.

I felt like I lived ten years. I felt like I was in a war for a long time. I stayed up till 3:30 in the morning for almost 2 months. I slept about 2 or 3 hours then went to work. I had to do 14 shifts straight without a day off due to staff shortage… I feel powerless to control my own life.

Lynn, 50-59 years, RN in long-term care (LTC) home, Chinese Canadian

For Grace, the mother of an adult son with developmental disabilities, the pandemic made her feel anxious about the future of her son.

COVID has also highlighted painful thoughts about what would happen to my son if anything should happen to me… COVID has made me think more about my own mortality and how much work is still left to be done to prepare my son [who is an adult with developmental disabilities].

Grace, female, 60-69 years, parent advisor in developmental disability sector, Canadian

Participant responses in this section illustrate the multilevel and cumulative impact of pandemic stressors experienced by HCPs that were often intricately connected to pre-existing sociocultural contexts and structural conditions of their lives, as in caring for children with disabilities or managing new and existing psychological distress.

## Discussion

### Principal Findings

In this paper, we applied a social ecological approach to make visible the complex synergistic stressors experienced by HCPs at home, at work, and in the community. The main findings of the study highlighted 5 key pandemic-related stressors experienced by HCPs that manifested at the personal, interpersonal, organizational, community, and societal levels. Taken together, the findings contribute to a more holistic understanding of the stressors and impacts experienced by HCPs during the first 2 waves of the COVID-19 pandemic in Canada.

Many of these stressors were shaped by pre-existing structural conditions. Although many of the workplace-related stressors for HCPs (eg, fear of getting infected, anxiety related to uncertainty, rapid changes in protocols, deployment, staff shortage) identified in this study are consistent with what has been reported in the emerging literature and news reports [13-15], this study offers insights drawn from participants’ qualitative reflections to contextualize and illustrate how workplace stressors interact with other stressors in the everyday lives of HCPs to negatively affect their physical, emotional, and mental health. We also illustrate how the experiences of individual HCPs are nested in organizational practices, which are shaped by structural influences and power relations in society.

At the personal and interpersonal levels, most of the participants were also caregivers before and after work, as daughters and caregivers of elderly family members, mothers of young children, or caregivers of loved ones living with special needs, disabilities, or serious illnesses. Yet, their personal life was intricately connected to their professional life and shaped by structural conditions. With or without the pandemic, unpaid and unrecognized caregiving in both the public and private realm is well recognized to be “invisible labor” that is also gendered and racialized [28,29]. In 2020, women made up over 80% of all workers in health care and social services in Canada [30]. Racialized immigrants, particularly adults from the Caribbean, Africa, and Southeast Asia, are overrepresented in nursing and health care support occupations, and over 58% of these adult immigrants with international training were overqualified for their employment [30]. Reflective of the statistics on the Canadian health care workforce, 85% of our 74 HCP participants self-identified as female, and about 50% identified as belonging to diverse ethnocultural communities beyond English and French. Most of the participants were nurses, social workers, LTC activity aides, counsellors, and therapists.

At the personal and professional levels, the hero discourse has posed many psychological dilemmas for HCPs. They are expected to be strong, invincible, all-enduring, and willing to sacrifice their personal well-being for the common good. When faced with the challenges of sustained long work hours, workplace chaos, physical and emotional exhaustion, and frequent experiences of vicarious trauma, this glorified expectation of self-sacrifice discourages HCPs from recognizing and embracing their limitations, leading to negative self-judgment, feelings of guilt, and a sense of incompetence that further compromise their mental health. In addition to workplace demands, they also have to attend to the physical, psychosocial, and spiritual needs of their family members and loved ones at home. When the hero discourse creates a spotlight solely on the professional identities and roles of HCPs, little attention is afforded to the multiple interdependent identities, experiences, and mental health needs of HCPs beyond their workplace. The results of this study enable us to highlight the seldom explored research gap of understanding the experiences of HCPs as both service providers and service users.

HCPs living with elderly family members or family members with weakened immune systems often function as protective agents as well as caregivers at home. During pandemic lockdowns, HCPs who are family caregivers of children or adults living with disabilities and special needs have to carry out specialized care, and the boundary between family caregiving and service provision becomes blurred. The required efforts to perform specialized care at home have created a double-bind paradox in which they have become unpaid service providers in their personal lives, while also having to maintain their highly demanding health care provision role at work. The multilevel intersecting stressors had taken a toll on the physical and mental health of HCPs, resulting in sleep disturbance, bodily pain, anxiety, anger, emotional exhaustion, burnout, and even suicidal ideation. Indeed, the Registered Nurses Association of Ontario (RNAO) Work and Wellness Survey [31] showed that 60% of respondents reported very high to high level of stress.

At the organizational level, the hero discourse has also created additional challenges for HCPs. Expressions of concern about workplace pandemic protocols, staff deployment, and shortage of protective equipment as well as questions about organization transparency may be dismissed as unimportant or a sign of individualistic selfishness. As indicated by some study participants, the hero discourse does not address HCPs’ frustration and distrust toward policy makers and institutions. Decades of neoliberal practices of austerity, decentralization, privatization, underfunded regulatory regimes, a culture focused on financial efficiency, and increasing controlled and standardized care work in the health and social care sectors have contributed to this distrust [28,32], which is heightened by the chaos, uncertainty, and unprecedented demands of the pandemic. Furthermore, despite making up over 80% of the health care workforce, there are disproportionately fewer women and racialized HCPs in leadership and decision-making positions within the health care system [33]. These structural inequities may help to explain the distrust and the sense of powerlessness expressed by participants. In addition, although established evidence shows that empowerment work environments and meaningful engagement of staff in decision-making are critical mitigating strategies to reduce burnout among HCPs [34,35], policy makers within the health care system have failed to address the challenge of burnout, leaving HCPs at increased vulnerability to burnout and distress during the pandemic [36]. Indeed, 13% of RNs aged 26 years to 35 years reported in the RNAO Work and Wellness Survey that they were very likely to leave the profession after the pandemic [31].

At the community level, many HCPs also experience moral distress [37] associated with a collective visceral resonance of fear, grief, empathy, stress, and anger as they witnessed the devastating impact of COVID-19 (death, loss, social isolation, xenophobia, and racism) on their patients, service users, co-workers, as well as vulnerable groups in the community. For some HCPs, the experience of moral distress intensified during the pandemic lockdowns when medicating children with behavioral challenges (attention deficit hyperactivity disorder, autism spectrum disorder) seemed to be the only viable option to prevent mental health crises of the affected families. For others, the frequent witnessing of patients “dying alone” and having to comfort them with “the touch of a gloved hand” are poignant reminders that the pandemic is not merely a communicable disease that threatens life and brings death; it is an unprecedented phenomenon that disrupts our taken-for-granted sociocultural values and desired practices of dying with dignity, surrounded by loved ones, and comforted by human connections of touch. The witnessing and partaking in these difficult situations left some participants with a sense of helplessness. The so-called pandemic new normal is anything but normal when services are shaped by the fast-changing protocols across the education, community care, and public health sectors, in which care work is deeply rooted in shared humanness between the care providers and care recipients [38,39].

At the societal or public policy level, the contributions of HCPs were not valued. During the COVID-19 pandemic, HCPs (especially nurses) have been hailed as “unsung heroes” by politicians, the mass media, and the general public [17]. Although the hero discourse has raised public recognition of the important contributions of HCPs, it has not amounted to anything to improve the well-being and pay equity of HCPs. Since November 2019, HCPs in Ontario had been calling the government to repeal Bill 124 without any success. Bill 124 restricts the wages of HCPs (who are mostly women) to a maximum of 1% even though the inflation rate has been over 2% over a decade, and other frontline professionals such as police and firefighters (who are mostly men) are not subject to this bill [17]. Bill 124 illustrates that the hero discourse is merely an empty rhetoric that does not provide any support needed by HCPs. In the Work and Wellbeing Survey of over 2100 nurses, conducted by the RNAO in early 2021, 81% of the respondents reported receiving very poor to fair support from the government, and 59% reported receiving very poor to fair support from their employers [31].

Amid the challenges and demands at work, some participants expressed their desire and commitment to ensure quality care for their patients and service users. For some, the workplace struggles served as a catalyst in strengthening their interpersonal connections with colleagues. The “we are all in this together” spirit seemed to have provided them with a sense of stability amid all the chaos and struggles. Indeed, many participants expressed a deep sense of altruism or responsibility to provide instrumental, psychological, and emotional support to their peers and those affected by the pandemic. This altruistic desire to help others seemed to help give meaning to their stressful work. Thus, the hero discourse is paradoxical in that it functions both as a stressor and a protective anchor. However, altruistic contributions and sustained stressors are often accompanied by adverse health consequences as indicated in the previous paragraphs.

### Implications for Policy and Practice

Consistent with the results of this study, 2 decades of research on large-scale epidemics and pandemics have shown persistent and pervasive negative personal impacts on the mental health of HCPs, including increased anxiety, burnout, and posttraumatic stress symptoms [40-42]. In addition, there is established evidence to show that organizational factors like staffing shortage, high workload demands, high work stress, low teamwork, low supervisor support, and work-life imbalance contribute to burnout and high rates of turnover [43-45]. In the fourth quarter of 2020, the job vacancy rate in health and social care increased to 4.7% [46]. The negative impact of COVID-19 on the mental health of HCPs has likely intensified the job vacancy rate. Thus, during a pandemic, it is imperative for health and social care organizations to attend to the mental health and emotional needs of HCPs.

Since the mental health of HCPs during the pandemic was influenced by complex and dynamic interplay of personal, interpersonal, organizational, community, and societal factors, a multiprong approach is necessary to mitigate these complex stressors. The RNAO Work and Wellness Survey [31] showed that, while 60% of respondents reported very high to a high level of stress, only 8.6% used mental health support through their employers, and only 1.1% used the provincial helpline. Instead, 49.6% sought support from their spouse, family, and friends, and 28.5% sought support from their colleagues. These results suggest that structured peer support programs at work may be an important strategy to maintain mental health of HCPs during a pandemic. Other organizational support such as flexible scheduling, destigmatization of help-seeking, and free programs that support HCPs to develop self-awareness of stress, self-care, and stress reduction are important. Free programs that apply mindfulness, music therapy, relaxation techniques, online workout, peer support, and one-on-one counselling have been found to be effective in stress reduction [47-49].

Additionally, providing timely and effective multiway communication and feedback can enhance social relationships and foster strong social support networks for HCPs within their organizations. Frequent timely communication of accurate, clear, and accessible information about the pandemic with HCPs of all levels is critical. At the same time, mechanisms for HCPs to report concerns, ask questions, and provide feedback to changing protocols are critical. Organizations may benefit from the real-time insights and feedback from the staff to refine their changing protocols. Although it is often impossible for organizations to adopt all suggestions from all staff, thoughtful responses to questions and concerns can be constructive in building and maintaining a collective vision, especially during a period of uncertainty and rapid change. Using peer liaison leaders in every department can enhance effective communication, build trust, and promote a sense of belonging among staff.

Finally, at the societal or public policy level, pandemic preparedness needs to be underpinned by the principles of social justice and equity. Across Canada, over 80% of the COVID-19–related deaths occurred in LTC homes [50]. Low wages, scarce sick benefits, and part-time employment meant that many LTC staff were working in multiple LTC homes, and some might have continued to work while they were ill [50,51]. Thus, existing inequitable distribution of resources within the health care system (eg, hospital vs LTC facilities), inadequate regulation of LTC homes, pay inequity for nursing staff, and the lack of access to full-time employment experienced by racialized staff in the lower ranks of the HCP hierarchy must be addressed.

### Limitations

In this study, we engaged participants who are reflective of the HCP workforce in Canada (ie, 85% self-identified as female), as well as ethnoracially diverse participants reflective of the Greater Toronto Area (close to 50% self-identified as belonging to visible minority groups). We have drawn on the qualitative reflection of participants to provide insights on the multilevel, intersecting sources of stressors experienced by HCPs amid a global pandemic. However, there are some study limitations. Since we used convenience and snowball sampling in recruitment, the range of HCP participants we engaged with might be limited. Furthermore, the self-reflective method did not allow us to engage in more in-depth exploration of specific phenomena that might have emerged from follow-up questions. As this is a qualitative component of the study, we did not generalize our findings. Rather, we have provided important contexts about the sources of stressors in the different domains of the participants’ lives, which are useful to guide follow-up research, programs, and policies in the postpandemic period. To reduce possible biases of the researchers toward a particular perspective, we have presented our theoretical framework, engaged team members with different disciplinary perspectives, and kept an audit trail of our analysis process including transcripts of responses, coding, and notes or recordings documenting decisions made in the study.

In addition, this paper reports only on the results of the qualitative component of a broader intervention study. It focuses on the sources and contexts of stressors experienced by HCPs during the first 2 waves of the pandemic. It does not include results on the feasibility and outcomes of PACER, which are beyond the scope of this paper. However, the evaluation protocols of PACER have been published elsewhere [19]. The evaluation of PACER is currently underway to examine the effectiveness of PACER in reducing the psychological impact of stress, improving mental health outcomes, and promoting resilience but is beyond the scope of this paper.

### Conclusion

Insights from this study demonstrate that COVID-19 is not merely a communicable disease but is also experienced as a social and political phenomenon. The reflections of HCP participants based on their lived experiences were situated within a social ecological perspective to provide a broader, more holistic understanding that reveals how personal and interpersonal experiences are shaped by power relations at organizational and societal or policy levels. Applying a socioecological lens allows for multilevel analysis that provides a deeper awareness of pre-existing challenges in the health and social care systems that become magnified during the pandemic. From a public health standpoint, it is critical for government and policy makers to engage HCPs in postpandemic discussion and also respond to pre-existing structural issues that contribute to the mental health stressors of HCPs. Finally, interventions from the individual to public policy levels to address the identified stressors must extend beyond the workplace identities and roles of HCP but to also include multidimensional aspects of their lives and consider home, community, and societal contexts. Thus, areas for future research could explore stressors according to the different types of job among HCPs, as well how stressors are experienced by HCPs from different identities, for example, deeper exploration of the stressors on HCPs from racialized or gendered identities and how they are impacted by these stressors.

## Abbreviations

ACE: Acceptance and Commitment to Empowerment
ACT: Acceptance and Commitment Therapy
AMO: Academic Medical Organization
CRCC: Canada Research Coordinating Committee
GEP: Group Empowerment Psychoeducation
HCP: health care providers
LTC: long-term care
MSH-UHN: Mount Sinai Hospital – University Health Network
NFRF: New Frontiers in Research Fund
PACER: Pandemic Acceptance and Commitment to Empowerment Response
PPE: personal protective equipment
RN: registered nurse
RNAO: Registered Nurses Association of Ontario
RPN: registered practical nurse
